# Infected Superior Mesenteric Artery Aneurysm following *Streptococcus gallolyticus* Bacteremia Associated with Cecal Cancer: A Case Report

**DOI:** 10.3400/avd.cr.26-00139

**Published:** 2026-10-07

**Authors:** Shintaro Kikuchi, Sosei Kuma, Kazuomi Iwasa, Kippei Ohgaki, Toshihiro Onohara

**Affiliations:** 1 Department of Vascular Surgery, Kyushu Central Hospital of the Mutual Aid Association of Public School Teachers, Fukuoka, Fukuoka, Japan; 2 Department of Surgery, Kyushu Central Hospital of the Mutual Aid Association of Public School Teachers, Fukuoka, Fukuoka, Japan

**Keywords:** mycotic aneurysm, visceral aneurysm, *Streptococcus bovis*

## Abstract

*Streptococcus gallolyticus* bacteremia is associated with colorectal malignancy and infective endocarditis, whereas infected superior mesenteric artery aneurysm (SMAA) is rare. A 94-year-old woman with cecal cancer developed *S. gallolyticus* sepsis that improved with intravenous antibiotics. Two months later, contrast-enhanced computed tomography revealed a newly developed 17-mm distal SMAA, suggesting an infected aneurysm secondary to the prior bacteremia. Because of the high risk of rupture, urgent aneurysmectomy and ileocecal resection were performed. This rare case highlights an infected SMAA as a potential vascular complication of *S. gallolyticus* bacteremia, particularly in patients with underlying colorectal malignancy.

## Introduction

Visceral artery aneurysms are rare, accounting for 0.1%–2% of all arterial aneurysms,^1)^ and superior mesenteric artery aneurysms (SMAAs) represent approximately 3.4% of these cases.^1)^ *Streptococcus gallolyticus* (*S. gallolyticus*) is well known for its strong association with colorectal neoplasia, bacteremia, and infective endocarditis.^2,3)^ To our knowledge, only 1 case of SMAA associated with *Streptococcus bovis* (*S. bovis*), now referred to as *S. gallolyticus* bacteremia due to colon diverticulitis, has been previously reported.^4)^ Herein, we report a rare case of an infected SMAA that developed shortly after *S. gallolyticus*-induced sepsis in a patient with newly diagnosed cecal cancer.

## Case Report

A 94-year-old woman was diagnosed with cecal cancer by colonoscopy performed for evaluation of anemia. She had a medical history of aortic valve replacement for severe aortic stenosis, ischemic heart disease, chronic heart failure, hypertension, hyperlipidemia, cerebral infarction, and a cerebral aneurysm. Immediately after the diagnosis of cecal cancer, she developed sepsis caused by *S. gallolyticus* and was initially treated with meropenem hydrate, followed by de-escalation to ampicillin sodium/sulbactam sodium, with subsequent clinical improvement. Contrast-enhanced computed tomography (CT) revealed no liver metastasis, lymphadenopathy, or SMAA (**Fig. 1A** and **1B**). Initially, the cecal cancer was managed conservatively because of her advanced age and comorbidities. Two months after the diagnosis, she was admitted with slurred speech and difficulty walking and was diagnosed with an acute cerebral infarction.

**Fig. 1:**
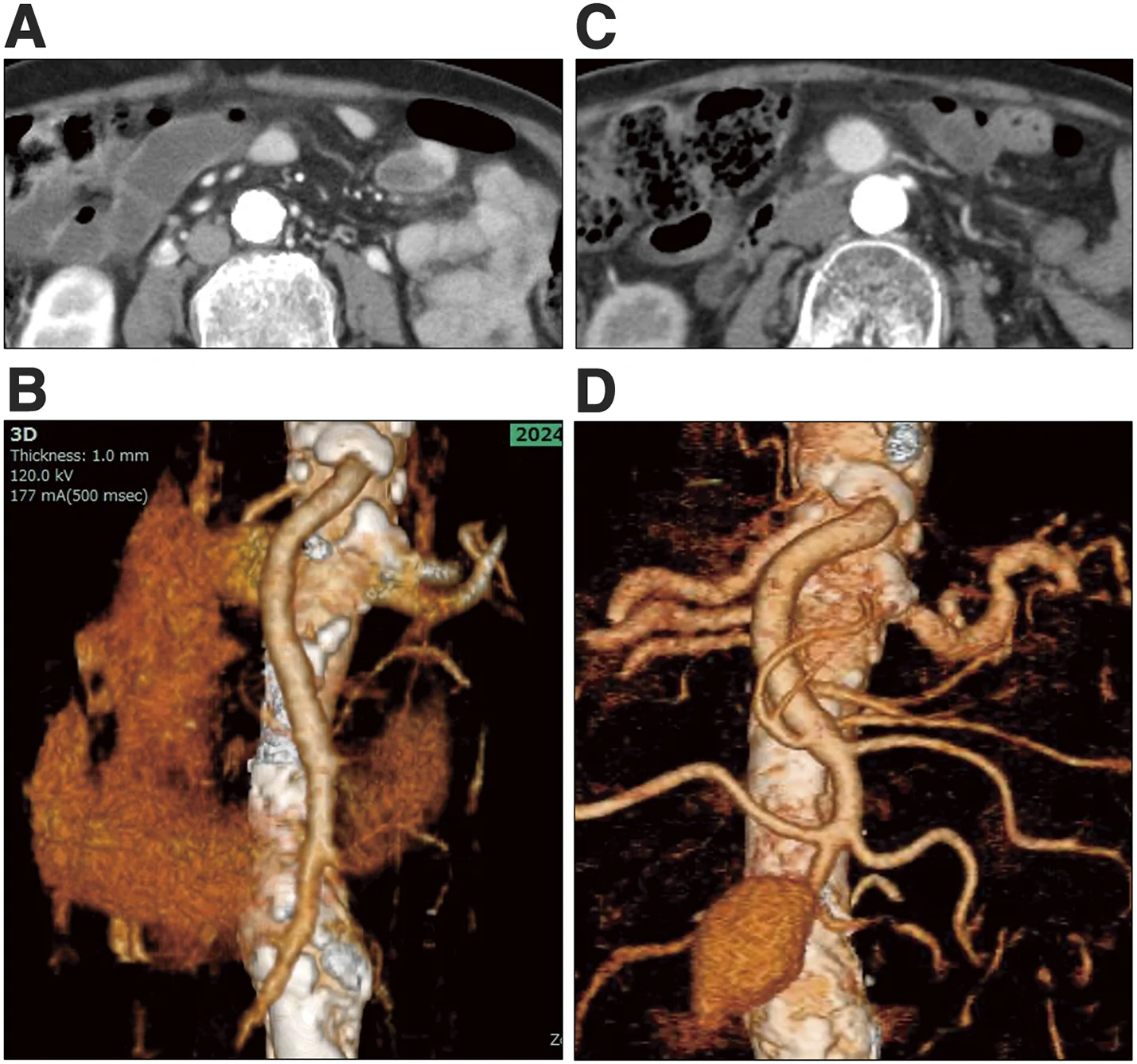
Computed tomography images 2 months before the surgery (**A**, **B**) and on readmission (**C**, **D**). There were no aneurysmal changes on the superior mesenteric artery (**A**, **B**). The superior mesenteric artery aneurysm was detected, and mild perivascular enhancement was observed (**C**). The aneurysm exhibited a fusiform morphology, enhancement of aneurysmal lumen was reduced (**D**).

On admission, laboratory examination revealed a white blood cell count of 7900/μL with neutrophil predominance (77.4%) and an elevated C-reactive protein level of 6.27 mg/dL. No bacterial growth was seen in the blood cultures. Contrast-enhanced CT revealed a fusiform aneurysm measuring 17 mm in short-axis diameter in the distal SMA near the bifurcation of the ileocolic artery, accompanied by mild perivascular fat stranding (**Fig. 1C** and **1D**). The aneurysmal lumen remained patent, whereas the distal portion was thrombosed. No significant atherosclerotic plaques suggestive of an embolic source were observed in the thoracic or abdominal aorta. Arteriography demonstrated an SMA aneurysm distal to the right colic artery. Inflow into the aneurysm was confirmed, whereas no apparent outflow vessel was identified (**Fig. 2A**). Transthoracic echocardiography revealed preserved left ventricular systolic function, with no regional wall-motion abnormalities, intracardiac thrombus, or valvular vegetation detected within the visualized range. A definitive diagnosis of infective endocarditis could not be established because transesophageal echocardiography was not performed. Diffusion-weighted brain magnetic resonance imaging showed scattered high-intensity lesions in the posterior limb of the left internal capsule, the left caudate nucleus, and the bilateral frontal and parietal cortices and white matter, consistent with acute cerebral infarction. Inflammatory markers were elevated on admission; therefore, intravenous ampicillin/sulbactam was administered. On hospital day 9, she was transferred to our department for surgical treatment of the SMAA and cecal cancer, and surgery was performed on hospital day 10.

**Fig. 2:**
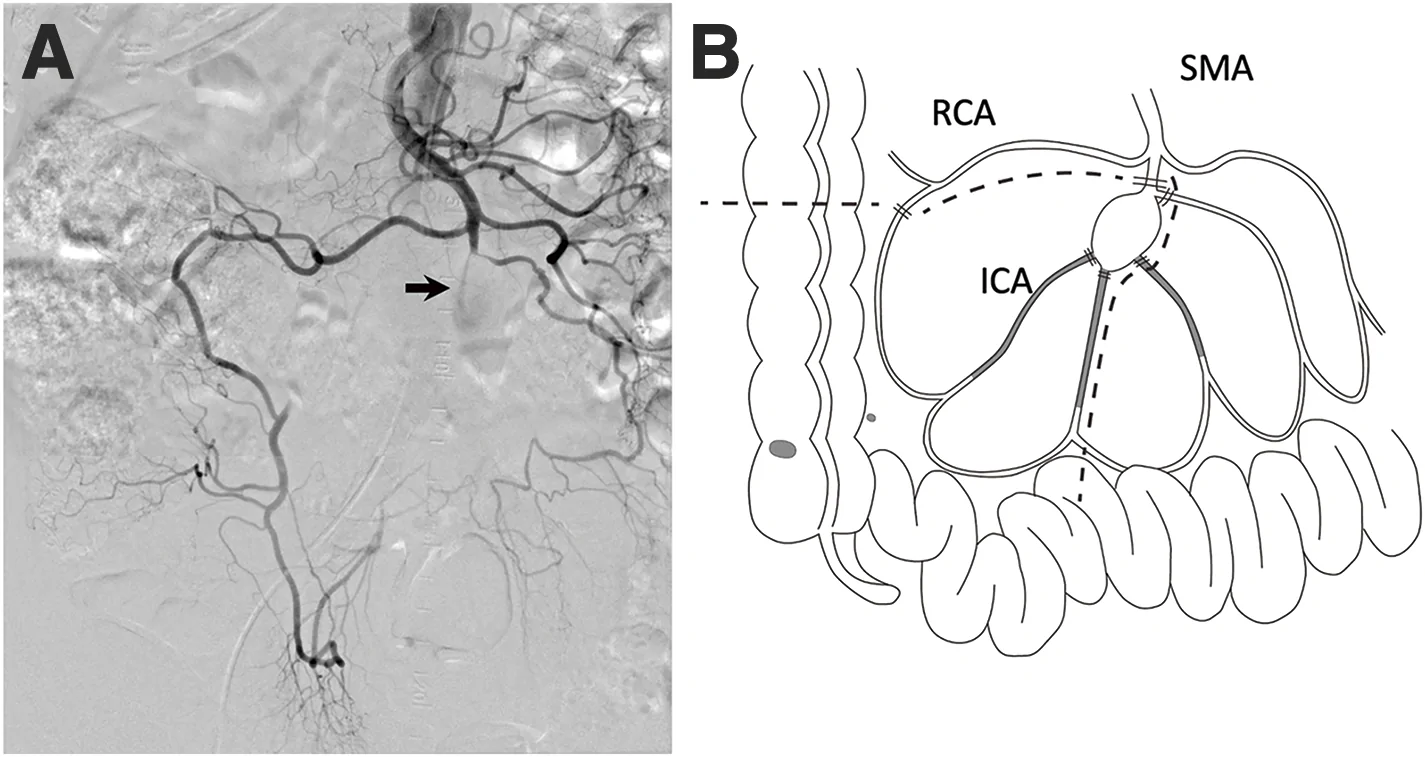
Preoperative arteriography (**A**) and operative schema (**B**). The aneurysm was located in the distal portion of the superior mesenteric artery. There was slight contrast enhancement within aneurysm lumen, no identifiable outflow vessel was visualized. After removing the SMAA, mesenteric division was subsequently performed on the colonic side, and the blood flow in the ileum was confirmed using Doppler ultrasonography and indocyanine green fluorescence imaging. The ileocecal resection was completed. Arrow indicates superior mesenteric artery aneurysm. ICA, ileocolic artery; RCA, right colic artery; SMA, superior mesenteric; SMAA, superior mesenteric artery aneurysm

Laparotomy was performed through a midline incision under general anesthesia with the patient in the supine position. Adhesions between the colonic and small intestinal mesentery were identified and carefully dissected to secure an adequate operative field. The SMA trunk, right colic artery, and ileal branches arising at the same level were dissected and encircled, followed by mobilization of the right hemicolon. The aneurysm was first resected, and mesenteric division was subsequently performed on the colonic side. After adequate ileal perfusion was confirmed intraoperatively by Doppler ultrasonography and indocyanine green fluorescence imaging, mesenteric division was performed on the ileal side (**Fig. 2B**). The ascending colon and ileum were transected using a Signia purple 60-mm stapling device (Medtronic, Minneapolis, MN, USA), and reconstruction was achieved by functional end-to-end anastomosis using the same device. The total operative time was 324 min, and intraoperative blood loss was 100 mL. Blood transfusion was not required. Histopathological examination of the aneurysm demonstrated marked intimal thickening with focal neutrophil infiltration, without significant atherosclerotic changes. The medial layer was thinned, with disruption and partial loss of the elastic lamina. The adventitia showed fibrous thickening accompanied by infiltration of lymphocytes and plasma cells. In the examined sections, no pseudoaneurysmal disruption of the arterial wall was identified, consistent with a true aneurysm (**Fig. 3**). No bacterial organisms were identified on histopathological examination.

**Fig. 3:**
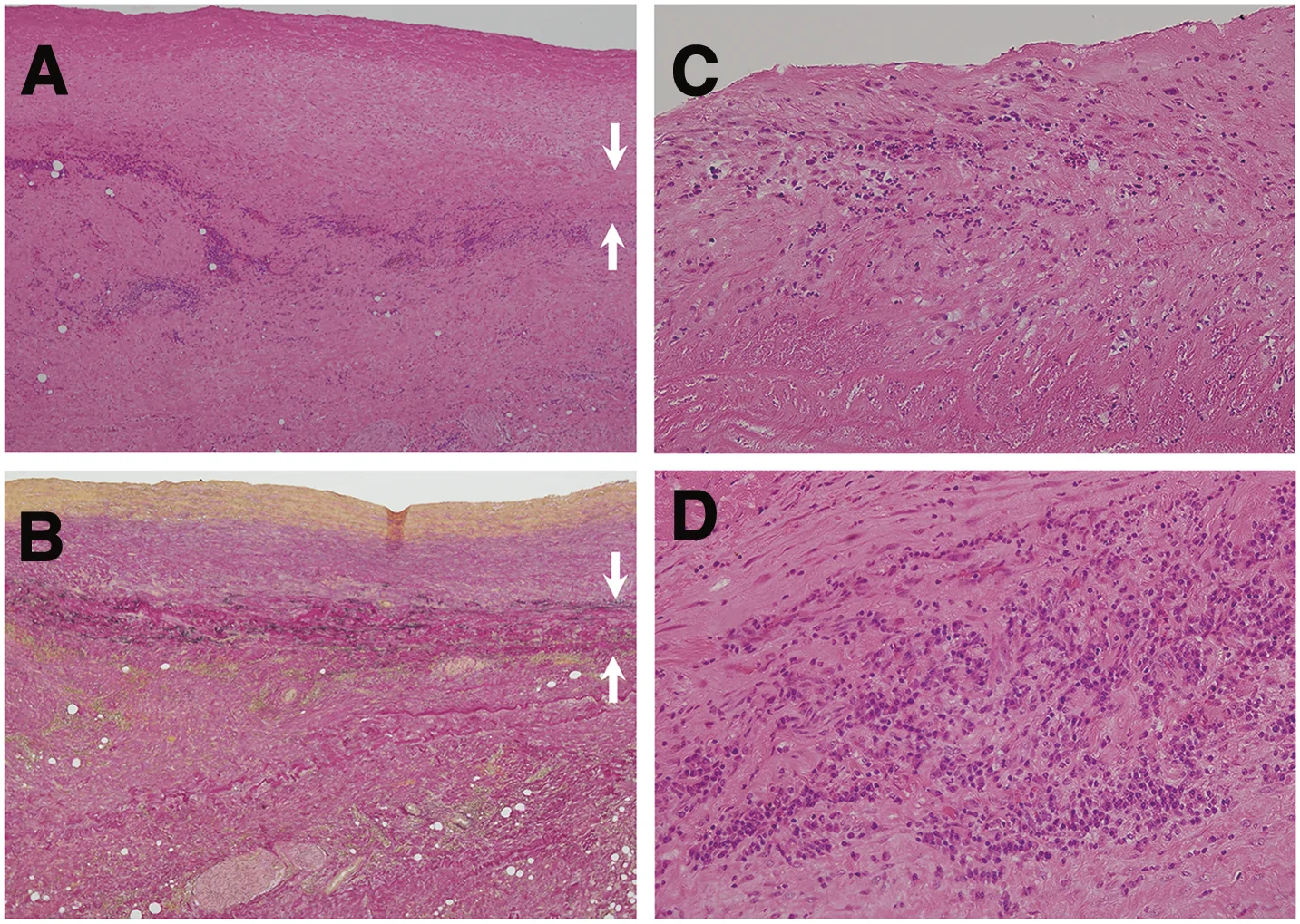
Pathological images of the superior mesenteric artery aneurysm. Histologic sections showing the marked intimal thickening, attenuation of the medial smooth muscle layer with disruption of the elastic laminae (**A**, **B**), neutrophil infiltration in intima (**C**), marked adventitial fibrosis in a multilayer pattern (**A**, **B**), and inflammatory infiltrate, including aggregations of lymphocytes (**D**). Arrows indicate media. (**A**) Hematoxylin–eosin stain (×40), (**B**) elastica van Gieson’s stain (×40), (**C**) hematoxylin–eosin stain (×200), (**D**) hematoxylin–eosin stain (×200).

Postoperative antimicrobial therapy with intravenous ampicillin/sulbactam was continued until postoperative day (POD) 14, with cefmetazole added until POD 2. On POD 6, thrombocytopenia developed, with a platelet count of 52000/μL, and the patient was transferred to the Department of Internal Medicine for further management. She was suspected to have idiopathic thrombocytopenic purpura, and her condition did not improve despite supportive treatment. She was subsequently transferred to a tertiary care center on POD 32 for specialized treatment and discharged 2 months later.

## Discussion

To our knowledge, this is the second reported case of an infected SMAA associated with *S. bovis*/*S. gallolyticus* bacteremia and the first to occur in the setting of cecal cancer without definitive evidence of infective endocarditis. *S. gallolyticus* is a Gram-positive coccus formerly classified as *Streptococcus bovis* biotype I^2)^ and is present in the intestinal flora of approximately 2.5%–15% of healthy individuals. Bacteremia due to *S. gallolyticus* is frequently associated with colorectal cancer or adenoma and is known to cause infective endocarditis.^2,3)^ Chronic inflammation mediated by cytokines induced by *S. gallolyticus* has been reported to contribute to colorectal tumorigenesis,^5)^ and 1 report indicated that 71% of patients with bacteremia caused by *S. gallolyticus* had concomitant colorectal cancer.^6)^ Conversely, the tumor mucosa may provide a favorable niche for *S. gallolyticus* colonization because of its altered metabolic environment, and impaired innate immune defense may facilitate bacterial entry into the bloodstream, leading to bacteremia or sepsis.^7)^

Infectious etiologies were historically considered to be a common cause of SMAA.^8)^ However, contemporary series in developed countries have shown infection to be a less common etiology, with most cases being degenerative.^9)^ In this case, the aneurysm developed within 2 months of documented *S. gallolyticus* bacteremia, strongly supporting an infectious etiology. Typical CT findings of infected aneurysms include saccular, multilobulated, or eccentric morphology, often accompanied by surrounding soft-tissue inflammation, fluid collection, or gas within or around the aneurysm. In contrast, although inflammatory changes were observed around the aneurysm in the present case, it was fusiform and did not exhibit the morphology typically observed in an infected aneurysm. Although microbiological confirmation from the aneurysmal tissue was unavailable, the aneurysm was not present on CT performed 2 months earlier and developed rapidly after documented *S. gallolyticus* bacteremia. Together with persistent inflammatory findings and histological neutrophilic infiltration, these findings strongly support the diagnosis of an infected aneurysm. Multifocal cerebral infarctions also raised concerns regarding septic embolization from occult infective endocarditis, although infective endocarditis could not be confirmed.

According to the current Society for Vascular Surgery guidelines, all infected visceral artery aneurysms warrant repair, regardless of their size, because of the substantial risk of rupture.^10)^ In recent years, reports of endovascular treatment for visceral artery aneurysms have increased, and when anatomically feasible, endovascular therapy is considered the first-line option.^10)^ In this case, because colectomy had initially been deferred because of the patient’s advanced age and comorbidities, and blood flow within the aneurysm was markedly reduced, coil embolization might have been an option despite the risk of intestinal ischemia. However, because the cecal cancer was considered the most likely source of sepsis, there were concerns regarding infection of coils, prosthetic valve infection, and aneurysm recurrence if the infected focus was left untreated. Therefore, simultaneous resection of the aneurysm and cecal tumor was performed. Although laparoscopic surgery might have been a less invasive option for frail patients, we performed open surgery because we anticipated difficulty in dissecting the tissue around the aneurysm. Angiography demonstrated no apparent outflow vessel from the aneurysm, suggesting that distal perfusion had already become dependent on collateral circulation. Because the colonic lesion was located within the perfusion territory of the aneurysm, vascular reconstruction was not required.

## Conclusions

We encountered a rare case of an infected SMAA that was presumed to have developed following *S. gallolyticus* bacteremia associated with cecal cancer. In patients with *S. gallolyticus* bacteremia, careful evaluation of colorectal lesions and the cardiovascular system, including possible vascular complications, is essential.
